# Di-μ-benzoato-κ^3^ *O*,*O*′:*O*′;κ^3^ *O*:*O*,*O*′-bis­[(benzoato-κ^2^ *O*,*O*′)(1,10-phenanthroline-κ^2^ *N*,*N*′)cadmium]

**DOI:** 10.1107/S1600536811022185

**Published:** 2011-06-18

**Authors:** Hong-Jin Li, Zhu-Qing Gao, Jin-Zhong Gu

**Affiliations:** a School of Chemistry and Biology Engineering, Taiyuan University of Science and Technology, Taiyuan 030021, People’s Republic of China; b Key Laboratory of Nonferrous Metal Chemistry and Resources Utilization of Gansu Province, College of Chemistry and Chemical Engineering, Lanzhou University, Lanzhou 730000, People’s Republic of China

## Abstract

The dinuclear title compound, [Cd_2_(C_7_H_5_O_2_)_4_(C_12_H_8_N_2_)_2_], lies on a crystallographic twofold axis. The Cd^II^ ions are connected by two bridging benzoate anions and each ion is seven-coordinated by five O atoms from three benzoate ligands and by two N atoms from 1,10-phenanthroline. The benzoate ligands adopt two different coordination modes, acting as bidentate and bridging tridentate ligands. The discrete neutral mol­ecules further extend their structure into a three-dimensional supra­molecular framework by inter­molecular π–π [inter­planar distances of 3.392 (4) Å] and C—H⋯π stacking inter­actions [H–mean plane = 2.567 (4) and 2.781 (4) Å].

## Related literature

For the structures and properties of cadmium compounds, see: Gu *et al.* (2007 ▶, 2011 ▶). For bond lengths and angles in related lead(II) compounds, see: Gu *et al.* (2011 ▶); Shi *et al.* (2008 ▶).
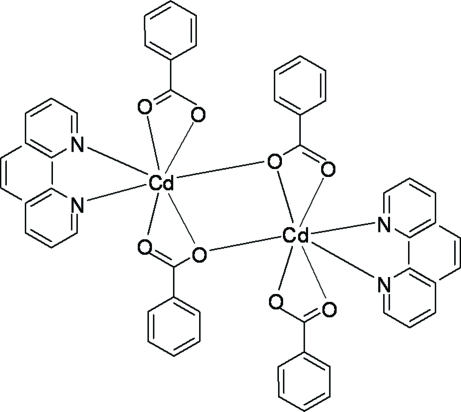


## Experimental

### 

#### Crystal data

[Cd_2_(C_7_H_5_O_2_)_4_(C_12_H_8_N_2_)_2_]
*M* *_r_* = 1069.65
Monoclinic, 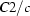

*a* = 21.90 (2) Å
*b* = 10.023 (11) Å
*c* = 20.52 (2) Å
β = 103.759 (10)°
*V* = 4376 (8) Å^3^
*Z* = 4
Mo *K*α radiation
μ = 1.04 mm^−1^
*T* = 296 K
0.28 × 0.26 × 0.24 mm

#### Data collection

Bruker APEXII CCD diffractometer
Absorption correction: multi-scan (*SADABS*; Bruker, 2004 ▶) *T*_min_ = 0.761, *T*_max_ = 0.789
15316 measured reflections
4068 independent reflections
3002 reflections with *I* > 2σ(*I*)
*R*_int_ = 0.037

#### Refinement

*R*[*F* ^2^ > 2σ(*F* ^2^)] = 0.040
*wR*(*F* ^2^) = 0.093
*S* = 1.12
4068 reflections
286 parameters
24 restraints
H-atom parameters constrained
Δρ_max_ = 0.80 e Å^−3^
Δρ_min_ = −0.62 e Å^−3^

### 

Data collection: *APEX2* (Bruker, 2004 ▶); cell refinement: *SAINT* (Bruker, 2004 ▶); data reduction: *SAINT*; program(s) used to solve structure: *SHELXS97* (Sheldrick, 2008 ▶); program(s) used to refine structure: *SHELXL97* (Sheldrick, 2008 ▶); molecular graphics: *DIAMOND* (Brandenburg, 2006 ▶); software used to prepare material for publication: *SHELXTL* (Sheldrick, 2008 ▶).

## Supplementary Material

Crystal structure: contains datablock(s) I, global. DOI: 10.1107/S1600536811022185/zq2105sup1.cif

Structure factors: contains datablock(s) I. DOI: 10.1107/S1600536811022185/zq2105Isup2.hkl

Additional supplementary materials:  crystallographic information; 3D view; checkCIF report

## Figures and Tables

**Fig. 1.**
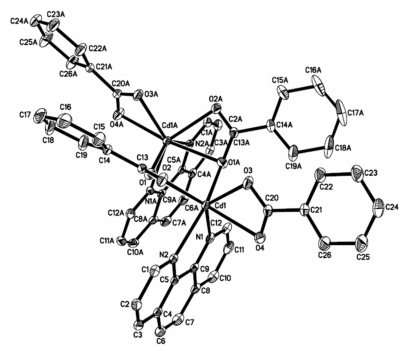
The molecular structure of the title complex showing the atom-labeling scheme and displacement ellipsoids drawn at the 30% probability level.

**Fig. 2.**
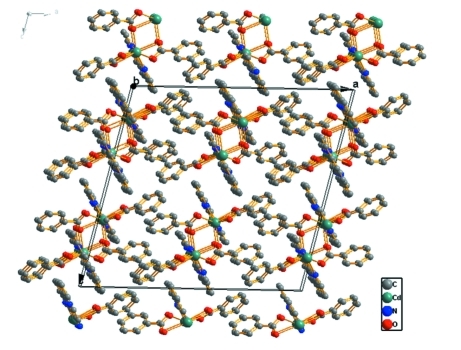
View of the crystal packing along the b axis, showing the three-dimensional framework structure of the title complex.

## supplementary crystallographic information

### Comment

Transition metal compounds have shown not only versatile architectures but also desirable properties, *e.g.* luminescent, magnetic, catalytic, and gas absorption and separation properties (Gu *et al.*, 2007; 2011). In order to extend our investigations in this field, we designed and synthesized one dinuclear cadmium(II) compound, [Cd_2_(C_7_H_5_O_2_)_4_(C_12_H_8_N_2_)_2_], and report its structure here.

The asymmetric unit of the title complex (Fig. 1) contains one Cd^II^ ion, two benzoate ligands, and one 1,10-phenanthroline molecule. Each Cd^II^ is seven-coordinate by five O atoms from three benzoate ligands, and two N atoms from 1,10-phenanthroline, and the coordination geometry around the Cd^II^ ion may be described as a distorted mono-capped trigonal prism. Two adjacent Cd^II^ units are connected by two bridging benzoate anions to generate a dinuclear complex. The dinuclear molecule lies on a crystallgraphic two-fold axis. The benzoate ligands adopt two different coordination modes acting as bidentate and bridging tridentate ligands.

The Cd—N bond distances of 2.347 (4) and 2.375 (4) Å and the Cd—O bond distances in the range of 2.265 (4)–2.493 (4) Å, are comparable to those reported for other Cd^II^—O and Cd^II^–N donor complexes (Gu *et al.*, 2011; Shi *et al.*, 2008).

In the crystal structure, π–π stacking interactions between adjacent 1,10-phenanthroline ligands are observed with interplanar distances of 3.392 (4) Å. Furthermore, adjacent benzene rings from benzoate ligands are involved in C—H···π stacking interactions (H—mean plane = 2.567 (4) Å). C–H···π stacking interactions between benzene rings from benzoate ligands and 1,10-phenanthroline ligands are also observed (H—mean plane = 2.781 (4) Å) . The discrete neutral molecules further extend their structure into a three-dimensional supramolecular framework by intermolecular π–π stacking interactions (Fig. 2).

### Experimental

A mixture of Cd(CH_3_COO)_2_.2H_2_O (0.14 g, 0.54 mmol), benzoic acid (0.12 g,1.0 mmol), 1,10-phenanthroline (0.11 g, 0.54 mmol), NaOH (0.04 g, 1.0 mmol), and water (10 ml) was stirred at room temperature for 15 min, and then sealed in a 25 ml Teflon-lined, stainless-steel Parr bomb. The bomb was heated at 433 K for 3 days. Upon cooling, the solution yielded single crystals of the title complex in *ca* 75% yield. Anal. Calcd for C_52_H_36_N_4_O_8_Cd_2_: C,58.39; H, 3.39; N, 5.24. Found: C, 58.73; H, 3.17; N, 5.63.

### Refinement

The H atoms were placed in geometrically idealized positions and constrained to ride on their respective parent atoms with C—H = 0.93 Å and *U*_iso_(H) = 1.2*U*_eq_(C).

### Crystal data

**Table:**

[Cd_2_(C_7_H_5_O_2_)_4_(C_12_H_8_N_2_)_2_]	*Z* = 4
*M**_r_* = 1069.65	*F*(000) = 2144
Monoclinic, *C*2/*c*	*D*_x_ = 1.624 Mg m^−^^3^
Hall symbol: -C 2yc	Mo *K*α radiation, λ = 0.71073 Å
*a* = 21.90 (2) Å	µ = 1.04 mm^−^^1^
*b* = 10.023 (11) Å	*T* = 296 K
*c* = 20.52 (2) Å	Block, colourless
β = 103.759 (10)°	0.28 × 0.26 × 0.24 mm
*V* = 4376 (8) Å^3^	

### Data collection

**Table:**

Bruker APEXII CCD diffractometer	4068 independent reflections
Radiation source: fine-focus sealed tube	3002 reflections with *I* > 2σ(*I*)
graphite	*R*_int_ = 0.037
φ and ω scans	θ_max_ = 25.5°, θ_min_ = 2.3°
Absorption correction: multi-scan (*SADABS*; Bruker, 1997)	*h* = −23→26
*T*_min_ = 0.761, *T*_max_ = 0.789	*k* = −12→12
15316 measured reflections	*l* = −24→24

### Refinement

**Table:**

Refinement on *F*^2^	Primary atom site location: structure-invariant direct methods
Least-squares matrix: full	Secondary atom site location: difference Fourier map
*R*[*F*^2^ > 2σ(*F*^2^)] = 0.040	Hydrogen site location: inferred from neighbouring sites
*wR*(*F*^2^) = 0.093	H-atom parameters constrained
*S* = 1.12	*w* = 1/[σ^2^(*F*_o_^2^) + (0.0278*P*)^2^ + 10.2493*P*] where *P* = (*F*_o_^2^ + 2*F*_c_^2^)/3
4068 reflections	(Δ/σ)_max_ = 0.001
286 parameters	Δρ_max_ = 0.80 e Å^−^^3^
24 restraints	Δρ_min_ = −0.62 e Å^−^^3^

### Special details

**Table:**

Geometry. All e.s.d.'s (except the e.s.d. in the dihedral angle between two l.s. planes) are estimated using the full covariance matrix. The cell e.s.d.'s are taken into account individually in the estimation of e.s.d.'s in distances, angles and torsion angles; correlations between e.s.d.'s in cell parameters are only used when they are defined by crystal symmetry. An approximate (isotropic) treatment of cell e.s.d.'s is used for estimating e.s.d.'s involving l.s. planes.
Refinement. Refinement of *F*^2^ against ALL reflections. The weighted *R*-factor *wR* and goodness of fit *S* are based on *F*^2^, conventional *R*-factors *R* are based on *F*, with *F* set to zero for negative *F*^2^. The threshold expression of *F*^2^ > σ(*F*^2^) is used only for calculating *R*-factors(gt) *etc*. and is not relevant to the choice of reflections for refinement. *R*-factors based on *F*^2^ are statistically about twice as large as those based on *F*, and *R*- factors based on ALL data will be even larger.

### Fractional atomic coordinates and isotropic or equivalent isotropic displacement parameters (Å^2^)

**Table:**

	*x*	*y*	*z*	*U*_iso_*/*U*_eq_	
C21	0.16729 (15)	0.0146 (4)	0.09716 (19)	0.0619 (13)	
C22	0.1880 (2)	−0.1080 (4)	0.1257 (2)	0.104 (2)	
H22	0.1688	−0.1463	0.1570	0.125*	
C23	0.2374 (2)	−0.1733 (4)	0.1075 (3)	0.124 (3)	
H23	0.2513	−0.2553	0.1266	0.149*	
C24	0.26614 (18)	−0.1160 (6)	0.0608 (3)	0.119 (3)	
H24	0.2992	−0.1597	0.0486	0.143*	
C25	0.2454 (2)	0.0065 (6)	0.0322 (2)	0.149 (4)	
H25	0.2646	0.0448	0.0009	0.179*	
C26	0.1960 (2)	0.0718 (4)	0.0504 (2)	0.116 (3)	
H26	0.1821	0.1539	0.0313	0.139*	
Cd1	0.025400 (15)	0.20368 (3)	0.167828 (17)	0.05215 (13)	
C1	−0.0718 (2)	0.3046 (6)	0.0284 (3)	0.0672 (13)	
H1	−0.0786	0.2135	0.0217	0.081*	
N2	−0.03302 (17)	0.3434 (4)	0.0840 (2)	0.0544 (9)	
C5	−0.0233 (2)	0.4760 (4)	0.0932 (2)	0.0547 (12)	
N1	0.04950 (18)	0.4302 (4)	0.1968 (2)	0.0561 (10)	
C12	0.0894 (2)	0.4697 (6)	0.2513 (3)	0.0718 (15)	
H12	0.1095	0.4058	0.2818	0.086*	
C8	0.0312 (3)	0.6576 (5)	0.1634 (3)	0.0673 (15)	
C6	−0.0421 (3)	0.7075 (6)	0.0599 (4)	0.0869 (18)	
H6	−0.0627	0.7702	0.0290	0.104*	
C10	0.0734 (3)	0.6964 (6)	0.2211 (4)	0.0846 (18)	
H10	0.0818	0.7865	0.2296	0.102*	
C11	0.1029 (3)	0.6042 (7)	0.2656 (3)	0.0856 (18)	
H11	0.1316	0.6296	0.3047	0.103*	
C4	−0.0541 (2)	0.5701 (5)	0.0467 (3)	0.0670 (14)	
C7	−0.0023 (3)	0.7484 (6)	0.1148 (4)	0.090 (2)	
H7	0.0041	0.8395	0.1221	0.107*	
C9	0.0200 (2)	0.5181 (5)	0.1524 (3)	0.0563 (12)	
C3	−0.0941 (3)	0.5224 (6)	−0.0112 (3)	0.0782 (16)	
H3	−0.1147	0.5820	−0.0438	0.094*	
C2	−0.1034 (2)	0.3905 (7)	−0.0209 (3)	0.0761 (15)	
H2	−0.1303	0.3580	−0.0597	0.091*	
O1	−0.06784 (16)	0.1836 (4)	0.21712 (17)	0.0731 (10)	
C13	−0.0884 (2)	0.0905 (5)	0.1775 (3)	0.0584 (12)	
O2	−0.06000 (17)	0.0531 (3)	0.1358 (2)	0.0846 (11)	
O4	0.10958 (18)	0.2070 (4)	0.1074 (2)	0.0885 (12)	
C20	0.1165 (2)	0.0874 (5)	0.1184 (3)	0.0603 (12)	
O3	0.08360 (17)	0.0266 (4)	0.1500 (2)	0.0852 (11)	
C14	−0.1497 (2)	0.0270 (6)	0.1776 (3)	0.0687 (14)	
C15	−0.1692 (3)	−0.0831 (7)	0.1401 (4)	0.106 (2)	
H15	−0.1437	−0.1201	0.1145	0.128*	
C19	−0.1870 (3)	0.0794 (9)	0.2161 (3)	0.120 (3)	
H19	−0.1735	0.1533	0.2430	0.144*	
C17	−0.2647 (5)	−0.0860 (17)	0.1749 (7)	0.204 (9)	
H17	−0.3045	−0.1215	0.1722	0.244*	
C16	−0.2269 (5)	−0.1412 (11)	0.1396 (6)	0.171 (5)	
H16	−0.2393	−0.2184	0.1148	0.205*	
C18	−0.2449 (4)	0.0220 (14)	0.2148 (5)	0.181 (6)	
H18	−0.2702	0.0571	0.2411	0.217*	

### Atomic displacement parameters (Å^2^)

**Table:**

	*U*^11^	*U*^22^	*U*^33^	*U*^12^	*U*^13^	*U*^23^
C21	0.049 (3)	0.066 (3)	0.072 (3)	0.001 (2)	0.016 (2)	−0.014 (3)
C22	0.086 (4)	0.078 (4)	0.163 (6)	0.021 (4)	0.060 (4)	0.003 (4)
C23	0.100 (5)	0.093 (5)	0.189 (8)	0.035 (4)	0.054 (5)	−0.006 (5)
C24	0.088 (5)	0.143 (7)	0.139 (7)	0.025 (5)	0.051 (5)	−0.037 (6)
C25	0.125 (7)	0.214 (10)	0.134 (7)	0.063 (7)	0.083 (5)	0.017 (7)
C26	0.106 (5)	0.157 (7)	0.102 (5)	0.042 (5)	0.060 (4)	0.023 (5)
Cd1	0.0478 (2)	0.04592 (19)	0.0689 (2)	0.00002 (17)	0.02607 (16)	−0.00061 (18)
C1	0.056 (3)	0.070 (3)	0.079 (4)	−0.006 (3)	0.025 (3)	−0.006 (3)
N2	0.049 (2)	0.052 (2)	0.069 (3)	−0.0022 (18)	0.027 (2)	−0.0026 (19)
C5	0.048 (3)	0.053 (3)	0.074 (3)	0.003 (2)	0.037 (3)	0.003 (3)
N1	0.050 (2)	0.057 (2)	0.068 (2)	−0.020 (2)	0.027 (2)	−0.019 (2)
C12	0.063 (3)	0.080 (4)	0.081 (4)	−0.018 (3)	0.034 (3)	−0.014 (3)
C8	0.072 (3)	0.044 (3)	0.103 (4)	−0.006 (3)	0.055 (3)	−0.009 (3)
C6	0.085 (4)	0.059 (4)	0.133 (6)	0.014 (3)	0.059 (4)	0.020 (4)
C10	0.095 (5)	0.062 (4)	0.114 (5)	−0.018 (4)	0.058 (4)	−0.027 (4)
C11	0.073 (4)	0.098 (5)	0.095 (4)	−0.035 (4)	0.039 (3)	−0.039 (4)
C4	0.059 (3)	0.063 (3)	0.094 (4)	0.007 (3)	0.047 (3)	0.011 (3)
C7	0.103 (5)	0.048 (3)	0.137 (6)	0.003 (3)	0.068 (5)	−0.003 (4)
C9	0.056 (3)	0.046 (3)	0.081 (4)	0.000 (2)	0.045 (3)	−0.004 (2)
C3	0.061 (4)	0.088 (4)	0.096 (4)	0.019 (3)	0.039 (3)	0.027 (4)
C2	0.057 (3)	0.101 (5)	0.070 (4)	0.001 (3)	0.015 (3)	0.007 (3)
O1	0.072 (2)	0.075 (2)	0.071 (2)	−0.0174 (19)	0.0138 (18)	−0.0037 (19)
C13	0.050 (3)	0.051 (3)	0.074 (3)	0.000 (2)	0.016 (3)	0.009 (3)
O2	0.072 (2)	0.061 (2)	0.133 (3)	−0.0092 (19)	0.049 (2)	−0.016 (2)
O4	0.088 (3)	0.075 (3)	0.117 (3)	0.027 (2)	0.054 (2)	0.019 (2)
C20	0.051 (3)	0.061 (3)	0.071 (3)	0.004 (3)	0.019 (2)	−0.011 (3)
O3	0.069 (2)	0.067 (2)	0.135 (3)	−0.0008 (19)	0.056 (2)	−0.009 (2)
C14	0.047 (3)	0.081 (4)	0.075 (3)	−0.011 (3)	0.010 (3)	0.018 (3)
C15	0.085 (5)	0.091 (5)	0.130 (6)	−0.035 (4)	−0.001 (4)	0.006 (4)
C19	0.060 (4)	0.207 (9)	0.099 (5)	−0.015 (5)	0.029 (4)	0.008 (5)
C17	0.084 (7)	0.311 (19)	0.182 (12)	−0.098 (10)	−0.036 (7)	0.124 (12)
C16	0.110 (8)	0.148 (8)	0.217 (13)	−0.077 (7)	−0.035 (7)	0.061 (8)
C18	0.060 (5)	0.363 (18)	0.126 (8)	−0.014 (7)	0.037 (5)	0.060 (9)

### Geometric parameters (Å, °)

**Table:**

C21—C22	1.3900	C8—C7	1.418 (8)
C21—C26	1.3900	C8—C9	1.429 (7)
C21—C20	1.481 (5)	C6—C7	1.317 (9)
C22—C23	1.3900	C6—C4	1.416 (8)
C22—H22	0.9300	C6—H6	0.9300
C23—C24	1.3900	C10—C11	1.350 (8)
C23—H23	0.9300	C10—H10	0.9300
C24—C25	1.3900	C11—H11	0.9300
C24—H24	0.9300	C4—C3	1.384 (8)
C25—C26	1.3900	C7—H7	0.9300
C25—H25	0.9300	C3—C2	1.344 (8)
C26—H26	0.9300	C3—H3	0.9300
Cd1—O3	2.265 (4)	C2—H2	0.9300
Cd1—O1^i^	2.331 (4)	O1—C13	1.249 (6)
Cd1—N2	2.347 (4)	O1—Cd1^i^	2.331 (4)
Cd1—O2	2.371 (4)	C13—O2	1.230 (6)
Cd1—N1	2.375 (4)	C13—C14	1.486 (6)
Cd1—O4	2.454 (4)	O4—C20	1.223 (6)
Cd1—O1	2.493 (4)	C20—O3	1.237 (6)
C1—N2	1.309 (6)	C14—C15	1.355 (8)
C1—C2	1.383 (7)	C14—C19	1.368 (8)
C1—H1	0.9300	C15—C16	1.388 (10)
N2—C5	1.352 (6)	C15—H15	0.9300
C5—C4	1.396 (7)	C19—C18	1.387 (10)
C5—C9	1.416 (7)	C19—H19	0.9300
N1—C12	1.306 (6)	C17—C16	1.344 (18)
N1—C9	1.320 (6)	C17—C18	1.364 (18)
C12—C11	1.396 (8)	C17—H17	0.9300
C12—H12	0.9300	C16—H16	0.9300
C8—C10	1.374 (8)	C18—H18	0.9300
			
C22—C21—C26	120.0	C10—C8—C9	117.9 (6)
C22—C21—C20	120.3 (3)	C7—C8—C9	118.5 (6)
C26—C21—C20	119.6 (3)	C7—C6—C4	121.5 (6)
C22—C21—Cd1	116.71 (19)	C7—C6—H6	119.3
C26—C21—Cd1	123.19 (19)	C4—C6—H6	119.3
C21—C22—C23	120.0	C11—C10—C8	120.3 (6)
C21—C22—H22	120.0	C11—C10—H10	119.8
C23—C22—H22	120.0	C8—C10—H10	119.8
C24—C23—C22	120.0	C10—C11—C12	118.4 (6)
C24—C23—H23	120.0	C10—C11—H11	120.8
C22—C23—H23	120.0	C12—C11—H11	120.8
C25—C24—C23	120.0	C3—C4—C5	117.2 (5)
C25—C24—H24	120.0	C3—C4—C6	123.5 (6)
C23—C24—H24	120.0	C5—C4—C6	119.3 (6)
C24—C25—C26	120.0	C6—C7—C8	121.9 (6)
C24—C25—H25	120.0	C6—C7—H7	119.1
C26—C25—H25	120.0	C8—C7—H7	119.1
C25—C26—C21	120.0	N1—C9—C5	120.8 (4)
C25—C26—H26	120.0	N1—C9—C8	120.4 (5)
C21—C26—H26	120.0	C5—C9—C8	118.8 (5)
O3—Cd1—O1^i^	89.55 (14)	C2—C3—C4	120.6 (5)
O3—Cd1—N2	125.34 (15)	C2—C3—H3	119.7
O1^i^—Cd1—N2	144.12 (13)	C4—C3—H3	119.7
O3—Cd1—O2	83.90 (15)	C3—C2—C1	118.2 (6)
O1^i^—Cd1—O2	108.88 (14)	C3—C2—H2	120.9
N2—Cd1—O2	85.43 (15)	C1—C2—H2	120.9
O3—Cd1—N1	133.60 (14)	C13—O1—Cd1^i^	136.0 (3)
O1^i^—Cd1—N1	79.49 (14)	C13—O1—Cd1	90.0 (3)
N2—Cd1—N1	70.31 (15)	Cd1^i^—O1—Cd1	103.66 (14)
O2—Cd1—N1	142.34 (13)	O2—C13—O1	121.0 (5)
O3—Cd1—O4	53.83 (13)	O2—C13—C14	118.5 (5)
O1^i^—Cd1—O4	110.26 (15)	O1—C13—C14	120.4 (5)
N2—Cd1—O4	88.04 (14)	C13—O2—Cd1	96.3 (3)
O2—Cd1—O4	120.44 (15)	C20—O4—Cd1	88.2 (3)
N1—Cd1—O4	87.97 (13)	O4—C20—O3	121.2 (5)
O3—Cd1—O1	123.30 (13)	O4—C20—C21	119.9 (5)
O1^i^—Cd1—O1	75.51 (14)	O3—C20—C21	118.9 (5)
N2—Cd1—O1	89.38 (13)	C20—O3—Cd1	96.8 (3)
O2—Cd1—O1	52.60 (13)	C15—C14—C19	119.4 (6)
N1—Cd1—O1	97.48 (12)	C15—C14—C13	120.7 (6)
O4—Cd1—O1	172.80 (13)	C19—C14—C13	120.0 (6)
N2—C1—C2	124.1 (5)	C14—C15—C16	120.6 (9)
N2—C1—H1	117.9	C14—C15—H15	119.7
C2—C1—H1	117.9	C16—C15—H15	119.7
C1—N2—C5	117.5 (5)	C14—C19—C18	119.9 (9)
C1—N2—Cd1	126.1 (3)	C14—C19—H19	120.1
C5—N2—Cd1	116.4 (3)	C18—C19—H19	120.1
N2—C5—C4	122.3 (5)	C16—C17—C18	120.1 (10)
N2—C5—C9	117.6 (4)	C16—C17—H17	120.0
C4—C5—C9	120.1 (5)	C18—C17—H17	120.0
C12—N1—C9	120.5 (4)	C17—C16—C15	120.0 (12)
C12—N1—Cd1	124.6 (4)	C17—C16—H16	120.0
C9—N1—Cd1	114.9 (3)	C15—C16—H16	120.0
N1—C12—C11	122.5 (6)	C17—C18—C19	120.0 (10)
N1—C12—H12	118.7	C17—C18—H18	120.0
C11—C12—H12	118.7	C19—C18—H18	120.0
C10—C8—C7	123.6 (6)		

Symmetry codes: (i) −*x*, *y*, −*z*+1/2.
